# Self-adaptive robot training of stroke survivors for continuous tracking movements

**DOI:** 10.1186/1743-0003-7-13

**Published:** 2010-03-15

**Authors:** Elena Vergaro, Maura Casadio, Valentina Squeri, Psiche Giannoni, Pietro Morasso, Vittorio Sanguineti

**Affiliations:** 1 University of Genoa, Department of Informatics, Systems and Telecommunications, Via Opera Pia 13, Genoa, Italy; 2 Italian Institute of Technology, Via Morego 30, Genoa, Italy; 3 ART Rehabilitation and Educational Centre, Piazza Soziglia 1/5, 16123 Genoa, Italy; 4 National Institute of Neuroscience, Turin, Italy

## Abstract

**Background:**

Although robot therapy is progressively becoming an accepted method of treatment for stroke survivors, few studies have investigated how to adapt the robot/subject interaction forces in an automatic way. The paper is a feasibility study of a novel self-adaptive robot controller to be applied with continuous tracking movements.

**Methods:**

The haptic robot Braccio di Ferro is used, in relation with a tracking task. The proposed control architecture is based on three main modules: 1) a force field generator that combines a non linear attractive field and a viscous field; 2) a performance evaluation module; 3) an adaptive controller. The first module operates in a continuous time fashion; the other two modules operate in an intermittent way and are triggered at the end of the current block of trials. The controller progressively decreases the gain of the force field, within a session, but operates in a non monotonic way between sessions: it remembers the minimum gain achieved in a session and propagates it to the next one, which starts with a block whose gain is greater than the previous one. The initial assistance gains are chosen according to a minimal assistance strategy. The scheme can also be applied with closed eyes in order to enhance the role of proprioception in learning and control.

**Results:**

The preliminary results with a small group of patients (10 chronic hemiplegic subjects) show that the scheme is robust and promotes a statistically significant improvement in performance indicators as well as a recalibration of the visual and proprioceptive channels. The results confirm that the minimally assistive, self-adaptive strategy is well tolerated by severely impaired subjects and is beneficial also for less severe patients.

**Conclusions:**

The experiments provide detailed information about the stability and robustness of the adaptive controller of robot assistance that could be quite relevant for the design of future large scale controlled clinical trials. Moreover, the study suggests that including continuous movement in the repertoire of training is acceptable also by rather severely impaired subjects and confirms the stabilizing effect of alternating vision/no vision trials already found in previous studies.

## Background

During the last years a considerable effort has been devoted to the application of robots as aids to the treatment of persons with motor disabilities, as documented in recent systematic reviews [1]. These studies suggested that robot therapy may be effective in accelerating the recovery of stroke survivors.

On the other hand, stroke survivors perform arm movements with abnormal trajectories/kinematics. They might elevate the shoulder in order to lift the arm, or lean forward with the torso instead of extending the elbow when reaching away from the body. Use of such incorrect patterns may limit their ability to achieve higher levels of movement ability, and may in some cases lead to repetitive use injuries. A common technique adopted by physiotherapists in routine training in order to address these problems is to "demonstrate" to the subjects the correct movement trajectories by manually moving their hand through it. The underlying assumption is that the motor system of the subject can learn to replicate the desired trajectory by experiencing it. Smooth manual guidance of subject's limb may also enhance somatosensory input involved in cortical plasticity and reduce spasticity by smooth stretching.

Robotic guidance has been shown to improve motor recovery of the arm following acute and chronic stroke [2]. Indeed robots may help recovery in two different ways: as measuring devices and as 'artificial therapists'. In the first case robots are capable of detecting all aspects of movement and haptic interaction and thus are crucial tools for understanding the mechanisms underlying recovery. As 'artificial therapists', robots may be programmed to implement a variety of highly reproducible, repetitive, training protocols.

Moreover, by combining these two aspects it is possible to monitor subject's performance in order to change in real-time the assistance in an adaptative way. This adds two powerful features to robot therapy that should be exploited in a suitable way: 1) exercises should be tailored to the specific impairment patterns of each subject and 2) they should adapt to the changing performance level. As a matter of fact, the amount of force a subject can contribute to a movement varies widely across subjects, in relation with different impairment levels, and also within a single subject as recovery progresses. Moreover, the motor system tends to behave as a 'greedy' optimiser [2] which exploits the assistive forces generated by the robot in such a way to reduce the degree of voluntary control (and therefore muscle activation); as a consequence, an assistive strategy that maintains a constant level of assistive force throughout sessions would progressively depress voluntary control instead of promoting it.

An approach for accounting systematically for these problems may be called "triggered assistance" and it is routinely used in some commercially available systems: the idea is that for each trial (e.g. reaching a target presented on a computer screen) the robot is initially passive and starts applying an assistive force only later on, if "triggered" by some criterion of failure (e.g. amount of time, lack of progress, error size etc.), forcing the subject to complete the movement. Different versions of this concept have been investigated, also including mechanisms that change controller parameters based on previous trials [3]. However, "triggered assistance" has an intrinsic discrete nature, which usually tends to break down the movement into two parts, with a rather jerky transition from the subject-driven initiation to the robot-driven termination of the movements.

On the other hand, the common wisdom coming from field practice in rehabilitation (see for example [4]) suggests that when helping a subject to perform a movement the therapist should apply the minimal amount of manual assistance in order to facilitate the emergence of voluntary, purposive control patterns. Shortly phrased this can be formulated as an assist-as-needed principle [5] or minimal assistance strategy [6]. Although triggered-assistance can be considered as a kind of assist-as-needed paradigm, we think it lacks two crucial components: 1) smoothness throughout the whole human-robot interaction, and 2) high-compliance interaction, which has the purpose of increasing freedom and thus promoting deeper involvement of the stroke survivor in the re-education process. The main goal of the strategy is to provide the minimum level of assistance that can allow the subject to initiate the action, without forcing him/her to complete the movement: this is the prerequisite for increasing voluntary neuromotor activity and encouraging neural plasticity.

Recently, Wolbrecht et al. [5] proposed an adaptive control scheme based on the assist-as-needed paradigm that allows to automatically adapt assistance to task performance, while providing enough assistance to support task completion. The controller generates the forces that the impaired person cannot provide autonomously, so that the movement is as normal as possible. To do that, the controller uses a general model for neuromuscular output that is learned adaptively for each subject and the desired movement trajectory needs to be completely specified.

In this test-case study we carry out a preliminary evaluation of an adaptive scheme of assistance in which the desired trajectory is only partially specified, in order to leave more freedom to the subject. The figural part of the trajectory is shown on the screen, as a figure-of-eight on which the target to be tracked slides smoothly, with a speed profile that is sensitive to the user's performance. Also the assistive force is modulated by the tracking performance. Due to the fact that the task is intrinsically continuous and smooth and operates in a large workspace, we expect that it could naturally facilitate the emergence of large size, fluent coordinated movements. The minimally assistive strategy, already investigated for reaching movements [6,7] is implemented by means of an adaptive control architecture that integrates continuous-time control with intermittent control and performance evaluation and can operate in two conditions: with or without vision, i.e. with open or closed eyes.

## Methods

### Experimental setup

**Figure 1:**
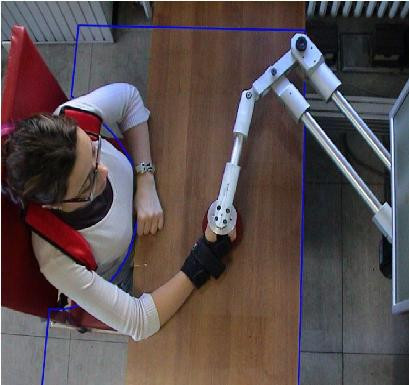
**Haptic robot Braccio di Ferro**. A view from above of a subject involved in the task.

We used a planar robotic manipulandum [8] characterized by low friction, low inertia, zero backlash, large elliptical workspace (80 × 40 cm) actuated by a pair of direct-drive brushless electric motors. Subjects sat on a chair, with their torso and wrist restrained by means of suitable holders, and grasped the handle of the manipulandum (fig 1) with their most affected hand. A light support was connected to the forearm to allow low-friction sliding on the horizontal surface of the table. Movements were restricted to the horizontal plane, with no influence of gravity. The position of the seat was also adjusted in such a way that, with the cursor pointing at the center of the workspace, the elbow and the shoulder joints were flexed about 90° and 45°, respectively, and the arm was kept approximately horizontal, at shoulder level. A 19" LCD computer screen was placed vertically in front of the subjects, about 1 m away, at eye level. In the vision task, the current position of the hand was continuously displayed, as a coloured 'car'. Target was also displayed as a round red circle (diameter 2 cm). The visual scale factor was 1:1. One may wonder if using a vertical LCD screen for displaying target and hand position, while the arm motion occurs in the horizontal plane, might be a problem for the patients. We could rule out this possibility, for the studied population of patients, because they immediately adapted to the experimental setup in the initial familiarization phase and answered in a positive way to a specific question by the physiotherapist asking if they understand the task and if they have any difficulty with the screen Moreover, the comparison between trials with open or closed eyes did not give any hint of a problem associated with the implicit visuo-motor mapping.

### Subjects

Ten subjects with chronic stroke (3 males, 7 females) volunteered to participate in this study (table 1). They were recruited among outpatients of the ART Rehabilitation and Educational Center - Genova. Inclusion criteria were (1) diagnosis of a single, unilateral stroke verified by brain imaging; (2) sufficient cognitive and language abilities to understand and follow instructions; (3) chronic conditions (at least 1 year after stroke), (4) stable clinical conditions for at least one month before entering robot therapy. Subjects ranged in age from 32 to 74 years (52.9 ± 14.99) with an average post-stroke time of 3.7 ± 1.95 years and with a majority of ischemic etiology (7/10). Three subjects had a history of left-hemisphere stroke; the others had right-hemisphere damage. As regards the impairment level (table 2), the majority of subjects (6/10) had a Fugl-Meyer score (arm section: FMA) smaller than 25/66. The other 4 subjects had a more moderate score (25<FMA<45). In any case, no subject was able to carry out the tracking task without robot assistance as we could verify in the preliminary familiarization session with the experimental setup.

**Table 1:** Anagraphical and clinical data of the patients.

Subject	Age	Sex	Disease duration	Etiology	Paretic hand
**S1**	74	M	4	I	L
**S2**	48	F	4	H	L
**S3**	36	F	4	I	R
**S4**	56	F	2	H	L
**S5**	32	F	3	I	L
**S6**	59	M	5	I	L
**S7**	71	F	4	I	R
**S8**	34	F	2	I	R
**S9**	57	F	8	H	L
**S10**	62	M	1	I	L

Age: years. Sex: Male/Female. Disease duration: years. Etiology: Ischemic/Hemorrhagic. Paretic hand: Left/Right.

The research conforms to the ethical standards laid down in the 1964 Declaration of Helsinki, which protect research subjects. Each subject signed a consent form that conforms to these guidelines.

The robot training sessions were carried out at the Neurolab of the Department of Informatics, Systems and Telematics of the University of Genoa, under the supervision of a physiotherapist, while a physiotherapist with more than twenty years of experience, selected the subjects, instructed them and evaluated the clinical scores.

### Experimental protocol and task

**Figure 2:**
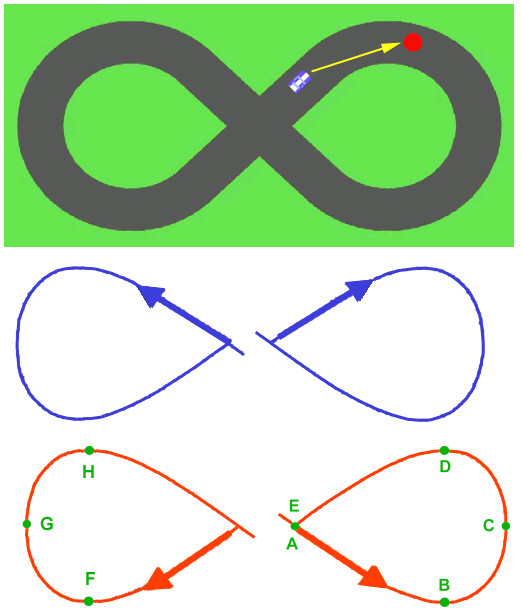
**Tracking task**. The top panel replicates the picture on the computer screen that includes the figure-of-eight path (black), the moving target (red circle), and the hand position (whitish car-shaped). The middle and bottom panels show the two tracking directions used in the experiments: clockwise-right/counterclockwise-left (blue), counterclockwise-right/clockwise-left (red). A - H are the eight control points used by the algorithm of performance evaluation.

The task consists of tracking a moving target that draws a figure-of-eight-shaped trajectory (length = 90 cm), according to the following law of motion:(1)

where *A *= 0.16 *m*, *B *= 0.07 *m*, *T *= 15 *s*. Therefore, it takes 15 s to complete the figure-of-eight, in the standard situation, i.e. if the target is not interrupted. This target formation law is consistent with the experimental analysis of handwriting movements [9], which shows that speed is strongly correlated with the curvature: speed is minimum where curvature is maximum and vice versa. In our case (see fig. 2 bottom panel) A, C, E are points of maximum speed (and minimum curvature): *v*_*A *_= *v*_*E *_= 8.9 cm/s, *v*_*C *_= 5.3 cm/s; B and D are points of minimum speed (and maximum curvature): *v*_*B *_= *v*_*D *_= 4.3 cm/s. These points, as well as the symmetric ones in the other half of the path (with a total of eight) are used as *control points *by the adaptive controller.

The position of the targets is presented simultaneously to the subjects in two sensory modalities:

• *visual*, by means of a circle on the computer screen;

• *haptic*, by means of an attractive force field directed towards the target.

The motion of the target is stopped if the error (distance between the target and the hand/robot position) exceeds 2 cm and it is resumed if the error re-enters the admissible error range. Chattering around the threshold is avoided by using a minimum duration after threshold crossing. The tracking duration of each turn is thus equal to the nominal duration of 15 s only if the error never exceeds the 2 cm threshold.

*Training sessions *are divided into *blocks*, each of them containing 10 turns around the figure: 5 turns with the sequence "clockwise-right/counterclockwise-left" plus 5 turns with the sequence "counterclockwise-right/clockwise-left" (figure 2). The nominal duration (for an ideal subject) is 10*15 = 150 s and the corresponding path length is 10*0.9 = 9 m. Each block of trials is carried out in one of two experimental conditions:

• *visuo-haptic condition *(VHC), in which the subject has vision of the hand position and the target on the computer screen and, at the same time, is provided with the haptic representation of the target direction by means of the attractive force field (from the hand to the moving target);

• *pure haptic condition *(PHC), in which the subject is blindfolded and only the robot-generated force field allows him/her to detect in which direction the target is moving.

VHC and PHC were alternated in the same session. Each session lasted no more than an hour and included a variable number of blocks, as a function of the impairment level: 18 in the ideal situation of perfect tracking. The therapy cycle included a number of sessions that ranged between 6 and 12 (see table 2).

**Table 2:** Clinical evaluation of the therapy.

Subject	No. of sessions	FMA pre	FMA post	ΔFMA	Ash
**S1**	11	4	8	4	3
**S2**	12	13	16	3	2
**S3**	10	25	31	6	1+
**S4**	12	36	38	2	1
**S5**	10	9	11	2	2
**S6**	10	22	23	1	3
**S7**	8	27	34	7	1+
**S8**	9	43	46	3	1
**S9**	6	44	48	4	1
**S10**	6	11	13	2	1+

**Mean ± SD**		23.4 ± 14.26	26.8 ± 14.6	3.4 ± 1.89	

FMA: Fugl-Meyer Arm section score (0-66), before (pre) and after (post) the robot therapy sessions. Ash: Ashworth score (0-4) before robot therapy (it did not change during therapy).

### Control architecture

**Figure 3:**
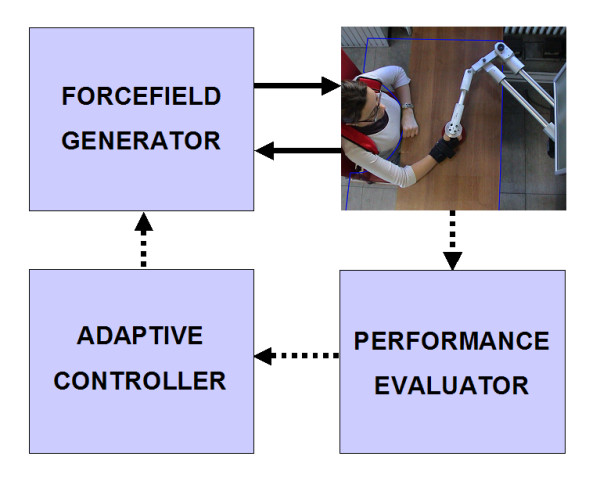
**Control scheme**. The *Force field generator *uses an impedance control scheme, with the direct drive of the robot actuators, in such a way to transmit to the handle a force vector computed as a function of the kinematic state of the robot (sampling frequency: 1 kHz). The *Adaptive Controller *modulates the gain of the force field as a function of the *evaluated performance*, according to a *non-monotonic *training protocol. Continuous vectors: continuous time control; Dotted vectors: intermittent control.

The control architecture, as indicated in figure 3, includes three main modules:

• *Force field generator;*

• *Performance evaluator;*

• *Adaptive controller*.

The *force field generator *uses an impedance control scheme:

1. the kinematic state of the robot (angles and angular velocities) is sampled at 1 kHz;

2. the state vector (position and velocity) is transformed from the joint space to the Cartesian space;

3. the instantaneous value of the force vector is computed as a function of the state, according to the desired structure of the force field (eq. 2 below);

4. the force vector is mapped from the Cartesian space to the joint space, using the transpose Jacobian matrix of the robot;

5. the computed torques are transmitted to the control units of the motors.

The force field used in the experiments has three different components:

• *Attractive or assistive component*: it is directed from the current position of the hand *x*_*H *_to the target *x*_*T*_, with an intensity that is proportional to the square root of the hand-target distance *d *= |*x*_*T *_- *x*_*H*_|;

• *Viscous component*, which is proportional to the arm speed and has the purpose of damping small amplitude, high frequency oscillations for the stabilization of the arm.

• *Repulsive component from a stiff surrounding wall*: the "wall" has an elliptic shape that surrounds the figure-of-eight and the repulsive force *F*_*W *_is unilateral and perpendicular to the wall.

Summing up, the force field is generated according to the following equation:(2)

where the viscous coefficient *B *is equal to 10 N/m/s, and the scale factor of the assistive field *K *is modulated by the adaptive controller. The force field generator is also in charge of moving the target according to Eq. 1 and stopping it if the distance between the hand and the target *E *= |*x*_*H *_- *x*_*C*_| is greater than a threshold *ET *= 0.02 *m*. In that case the controller waits for the subject to re-enter inside the error tolerance.

The *performance evaluator *updates a score by counting the number of times the control points are passed with a tracking error within tolerance. At the end of the current block of trials the evaluator performs two checks: it compares 1) the actual score with a threshold (a percentage of the maximum score) and 2) the total duration with another threshold (twice the nominal duration, which corresponds to a no-stop block). If both checks are positive, then the adaptive controller is instructed to reduce the gain *K *in the next block.

The *adaptive controller *modulates the gain *K *of the force field as a function of the evaluated performance in the previous block of the current session or in the last block of the previous session. At the beginning of a session, the controller retrieves the gain used in the last block of the previous session and applies a suitable increment, thus implementing a non-monotonic, inter-session adaptation strategy. In the following blocks the gain is decreased if both checks performed by the performance evaluator are positive, according to a monotonic intra-trial adaptation strategy. This mixture of non-monotonic and monotonic adaptation was applied successfully with reaching/hitting movements [6] and is motivated by the fact that any minimal assistance strategy must achieve a stable trade-off between performance accuracy, which would require a high assistance level, and task difficulty, which has an opposite requirement.

The controller, as well as the performance evaluator, is activated intermittently whereas the force field generator is activated continuously. In summary, the control architecture is characterised by the following pseudo-code:

Session_start:   set *K *= *K*_*last_session *_+ *ΔK*

Block_start:   set *SCORE *= 0 &*DURATION *= 0

Iterate:       for each *TURN *(1:*NT*) & each *CONTROL_POINT *(1:*NC*)

            compute *E *= |*x*_*H *_- *x*_*C*_|

            if *E *<*ET *then increment *SCORE*

            if *E *>*ET *then wait until *E *<*ET*

            update *DURATION*

         if *TOTAL_TIME *> 45 min then stop

         if *SCORE *>*ST *&*DURATION *<*DT *then K = *K *- Δ*K*

         go to Block_start

For the parameters that characterize the control algorithm (Δ*K*, *ST*, *DT*, *ET*, *NT*, *NC*) we used the following values, which were chosen empirically, by trial and error, in order to match the subject's requirements:

1. Δ*K *(gain increment/decrement): 3;

2. *ST *(score threshold): 75%;

3. *DT *(duration threshold): 2*(15*10) = 300 s;

4. *ET *(tracking error threshold): 0.02 m;

5. *NT *(number of turns for each block): 5+5 = 10;

6. *NC *(number of control points for each turn): 8.

The adaptive control strategy described above is intrinsically robust and avoids oscillations of the assistance that might occur in a continuous time adaptive scheme.

The initial values of the force field's gain *K *are selected before the first session as the minimum level capable to induce the initiation of movement of the paretic limb.

We should emphasize that, although the robot generates a force field that assists the subject in tracking the target, it does not impose the trajectory and/or the timing: unless a suitable degree of voluntary control is provided by the subject, the target cannot be pursued successfully. In other words, the black corridor that surrounds the figure-of-eight on the PC screen is only graphic and does not implies any active constraint by the robot.

Summing up, the temporal structure of the experiment control software is characterized as follows:

• Force field generation and impedance control: continuous time (sampling frequency 1 kHz);

• Virtual reality (visual and acoustic): continuous time (sampling frequency 100 Hz);

• Data acquisition: continuous time (sampling frequency 100 Hz);

• Adaptive control: intermittent, triggered by the completion of a block.

The control software is based upon Simulink/Matlab (Mathworks Inc). In particular the exercise protocol is specified as a finite-state machine, implemented by means of Stateflow (a standard Matlab tool). The virtual reality environment is implemented by means of the Virtual Reality Modeling Language (VRML), using Simulink's Virtual Reality toolset. The real time application is developed using a Simulink based fast-prototyping environment, RT-LabR_(Opal-RT Technologies Inc.).

### Data analysis

Hand position was measured from the 17-bit encoders of the motor with a precision better than 0.1 mm in the whole workspace. Hand speed (and subsequent derivatives) was estimated by using a 4th order Savitzky-Golay smoothing filter (with an equivalent cut-off frequency of ~6 Hz). The subjects' goal was to perform accurate and smooth tracking movements, thus we used two indicators that are not only task relevant, but, taken together, describe the overall subject performance during each trial:

1. *Movement arrest time ratio (MATR)*: mean value over a trial of the ratio between the time in which the hand stops (the speed is less than 20% of the mean speed) and the total duration of the movement. It measures the degree of segmentation of the tracking movements [10]. As training proceeds, this indicator should go down to 0. Qualitatively, this parameter expresses the subjective difficulty of the person in attempting to meet the task, thus including momentary stops of his/her movements or movements in wrong directions.

2. *Tracking error (TE)*: it is computed as the mean value of the distance of each point of the path from the theoretic path (the figure-of-eight trajectory). It is a measure of accuracy [11]; as training proceeds this indicator should go down to 0.

*MATR *is an indicator of smoothness and *TE *of accuracy. These indicators were averaged for each block and for each session.

### Statistical analysis

Although this paper is only a feasibility study and does not intend to evaluate the clinical efficacy of the proposed assistive method of robot therapy, we carried out a statistical analysis in order to have a preliminary estimate of the order of magnitude of the performance changes induced by the therapy sessions, including vision/novision effects. On this purpose, for each indicator, we ran an ANOVA with two factors: VISION (yes, no) and SESSION (first, last).

We also analysed, for each indicator, the difference between the values in the vision and no-vision conditions, with the purpose of ascertain whether the absolute value of this difference is reduced significantly during training. On this purpose, we ran a 1-way ANOVA.

## Results

### Overall effects

**Figure 4:**
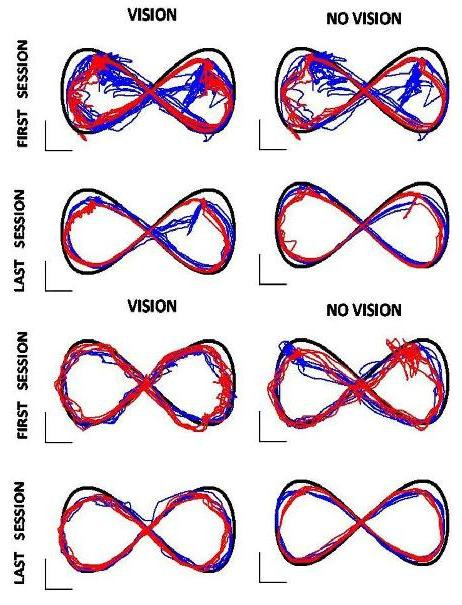
**Tracking trajectories**. Top panel is related to subject S1 who has a sever impairment level (FMA = 4). Bottom panel is related to subject S3 who is affected in a lighter way (FMA = 25). Blue line denotes the clockwise-right/counterclockwise-left sequence; Red denotes the counterclockwise-right/clockwise-left sequence. The black line represents the correct trajectory.

Figure 4 shows the general aspect of tracking trajectories at the beginning and the end of the treatment, for two subjects with different levels of impairment: S1 (FMA = 4), S3 (FMA = 25). This figure illustrates quite well that different stroke lesions can lead to quite different kinematic behaviours.

S1 (a male) has a great difficulty to track the target initially, as regards the farther ends of the nominal path in both the VHC ("vision") and PHC ("no vision") conditions: he can indeed approach those areas of the workspace, which require almost full extension of the arm, but is unable to produce the movement in a smooth way; thus he halts and can recover tracking only after several attempts. Please note that the level of assistance is not increased during such arrest times: the ability to get out of the blocking conditions is totally self-generated, although facilitated by the assistance scheme. At end of training the trajectories are generally smoother and show less halts.

S3 (a female) has a smaller difficulty to track, particularly in the VHC condition that does not exhibit any halting episode. At the end of training, however, the tracking performance appears to be smoother in the purely haptic condition than in the vision-dominated condition.

**Figure 5:**
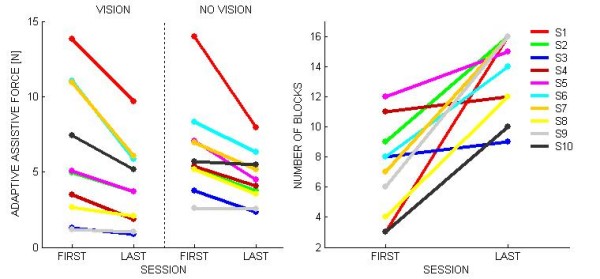
**Evolution of robot assistance during training**. The left panel shows the evolution over the training process (sessions 1-10) of the average assistance force for each session, in the two experimental conditions (*vision *and *novision*). The right panel shows the increase of the number of blocks per session that could be fully completed by all the subjects in the nominal session duration (45 min).

The left panel of figure 5 shows, for all the subjects, the reduction of the haptic assistance over the training sessions, in the two experimental conditions. The level of assistive force in the first session ranges between 1 N and 15 N and is generally higher for more severe subjects. The statistical analysis shows a significant decreases over sessions of the level of assistive force for the combined set of experiments (F(1,9) = 13.231 p = 0.00542)). In the no vision condition it is apparent that the assisting force does not go down the 3-4 N level and this is consistent with acknowledged perceptual thresholds of the proprioceptive channels.

The right panel of figure 5 shows that for all the subjects the number of blocks, performed in the canonic time window, increased with training. This suggests that the subjects became better and better in tracking the target with lower and lower robot assistance. This trend is further analyzed by looking at the performance indicators.

### Evolution of the indicators

**Figure 6:**
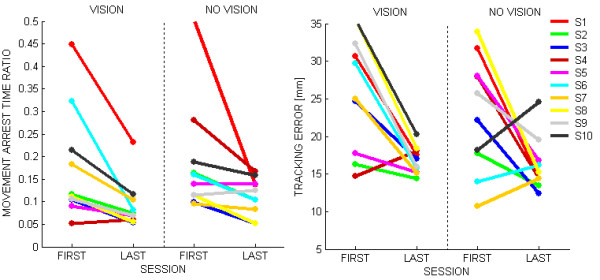
**Evolution of the performance indicators**. Left panel: Movement Arrest Time Ratio; Right panel: Tracking error.

Figure 6 shows the evolution of the indicators described in the methods, namely *MATR *(movement arrest time), and *TE *(tracking error).

In both cases, the statistical analysis showed a significant decrease between the beginning and the end of the treatment: (F(1,9) = 9.05 p = 0.015) for *MATR *and (F(1,9) = 25.43 p = 0.0007) for *TE*. This means that there was a measurable effect of treatment for all subjects as regards smoothness (*MATR*) and accuracy (*TE*).

**Figure 7:**
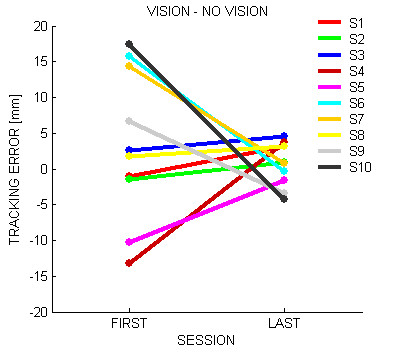
**Vision Novision convergence**. Difference between the accuracy in the *vision *and *no vision *conditions. A negative value means that subjects perform better in the vision condition; a positive value corresponds to the opposite situation.

Finally we compared the *accuracy *of the performance with and without vision. (Figure 7). At the beginning of the treatment, some subjects show better performance in the vision condition (S4, S5), other in the no vision condition (S6, S7, S9, S10) and the remaining subjects (S1, S2, S3, S8) show a negligible difference. At the end of training, however, for the accuracy indicator the difference decreased to a level that is statistically equivalent to 0 (F(1,9) = 7.4079 p = 0.02354). This suggests an equalization of the performance between the VHC and PHC conditions.

### Clinical results

Across sessions the subjects showed a significant improvement in the modified FMA scale, without any increase of the Ashworth score, as shown in Table 2. In particular, we found a significant (p = 0.0002) increase in the FMA score, from 23.4 ± 14.26 to 26.8 ± 14.6, corresponding to 3.4 ± 1.89 on average.

## Discussion

Although the reported pilot study shows a consistent and significant improvement in the coordination and functional parameters of the participating stroke survivors, no firm conclusion can be drawn at this time because it does not satisfy many of the requirements of controlled clinical trials. However, in the spirit of a feasibility study, the purpose was rather to acquire some empirical knowledge on a few crucial points that are relevant for the design of novel, effective protocols of robot-subject interaction:

• Stability of the self-adaptive minimal assistance strategy;

• Triggered vs. continuous assistance;

• Rationality of non-monotonic assistance;

• Range of impairment that can be addressed.

The stability of the proposed interaction strategy is apparent if we consider the evolution of the level of the assistive force, which is characterized by a consistent decrease in all the experimental conditions. This is remarkable because the force level is not imposed but is the result of two actions: 1) the modification of the gain of the force field carried out by the robot controller and 2) the modification of the motor control patterns performed by the subject. Thus, the results are consistent with the conclusion that the proposed interaction scheme can promote a synergy between adaptability of the robot and plasticity of the brain, i.e. an optimal trade-off between robot-influenced performance level and brain-driven voluntary control.

Furthermore, we suggest that this kind of synergy can be achieved as a consequence of two main elements:

1. *Continuity *of the robot-patient interaction: the force-field generator provides a continuous and smooth force field that obviously promotes smooth motor patterns. Although smoothness per se is not a functional indicator of motor recovery, it has been shown that movement smoothness can promote recovery from stroke [10]. For this reason we believe that what we called "triggered assistance" is not appropriate because it tends to break down the smoothness of the robot-subject interaction.

2. *Stability *of the interaction parameter over the current task (turn or block in our case). A continuous mechanism of modification of the interaction parameters, e.g. the gain of the force field, would introduce an element of randomness/instability in the haptic interaction that is likely to be detrimental for the ordered acquisition and mastering of new control patterns.

The implemented interaction mechanism combines continuity within trials with adaptive modification across trials and across sessions. We suggest that this is crucial for allowing the proposed system to be effective with subjects characterized by widely different impairment levels. The reported experiments are consistent with this view (the FMA score ranges between 4 and 44 in the population of subjects), although this has to be confirmed by a much larger population.

The efficacy of the self-adaptive mechanism for a large range of impairments is also enhanced by the fact that the use of a continuous force-field, not a triggered action, is at the same time *assistive *(it facilitates the acquisition of the target) and *informative *(it lets the subject know, in real-time, where the target is also in the absence of vision). For slightly impaired subjects this kind of additional information may be almost irrelevant but for more severe ones it may be crucial for the reacquisition of internal control models. Again, this possibility would become impossible with a triggered mechanism of assistance. For severe patients, who have a more complex task in building/rebuilding internal control models, the predominance of vision is useful for helping to carry out the current movement but is a barrier for overcoming badly-adapted compensatory patterns. The alternation of vision and no vision blocks is likely to be a beneficial challenge for severely impaired subjects: it is difficult but doable. We also suggest that a contribution in this direction (widening as much as possible the range of impairment levels) comes from the non-monotonic decrease of the field gain. This avoids the possible frustration of severely impaired subjects at the beginning of a session, a few days after the previous one. The extra assistance that is allowed in the first block of a session allows these subjects to avoid remaining stuck in a too difficult situation.

Whatever performance indicator is used, the difference between vision and non vision conditions decreases across sessions. This is clearly a positive clinical sign, because it suggests a recalibration of the sensory channels, as an effect of training, which is crucial for carrying out purposive motor actions,. In any case, it is remarkable that the subjects were indeed capable of operating only on the basis of proprioceptive cues.

The subjects of this feasibility study exhibit a significant improvement in the modified FMA scale. The clinical score increased: 3.4 ± 1.89 on average. This result is in line with previous studies [1], which report an average improvement of 3.7 ± 0.5.

## Conclusions

The results of this preliminary study provide detailed information about the stability and robustness of the proposed adaptive controller of robot assistance that could be quite relevant for the design of future large scale controlled clinical trials. The results also demonstrate that personalization of robot therapy by means of suitable self-adaptive interaction strategies is practical and support the assumption that personalization might be a crucial element for achieving optimal assistance. We also believe that personalization of robot assistance is a pre-requisite for overcoming the barrier between improvements in the coordination/control parameters and functional achievements in activities of daily life. Moreover, the study shows that including continuous movements in the repertoire of training protocols is promising because it is well accepted also by rather severely impaired subjects and enriches the range of movement directions that are implicitly trained. The stabilizing effect of alternating vision/novision trials, already found in previous studies, is further confirmed, emphasizing the need of integrating movement and proprioception training in the same experimental paradigm.

## Competing interests

The authors declare that they have no competing interests.

## Authors' contributions

The overall design of the experiments was agreed by all the authors after extensive discussions. E.V., M.C., and V.Sq. implemented the protocol, carried out the experiments, and analyzed the data. P.M. drafted the manuscript. P.G., who is a physiotherapist, selected the stroke subjects, instructed them and evaluated the clinical scores. V.S. defined and performed the statistical analysis.

All authors read and approved the manuscript.
